# Outcomes and Functional Deterioration in Hospital Admissions with Acute Hypoxemia

**DOI:** 10.3390/arm92020016

**Published:** 2024-03-06

**Authors:** Susanne Simon, Jens Gottlieb, Ina Burchert, René Abu Isneineh, Thomas Fuehner

**Affiliations:** 1 Department of Respiratory Medicine and Infectious Diseases, Hannover Medical School, 30625 Hannover, Germany; gottlieb.jens@mh-hannover.de; 2 German Center for Lung Research (DZL), 30625 Hannover, Germany; 3 Department of Respiratory Medicine, Siloah Hospital, 30459 Hannover, Germany; ina.burchert@krh.de (I.B.); thomas.fuehner@krh.de (T.F.); 4 Department of Gastroenterology, Hepatology and Endocrinology, Hannover Medical School, 30625 Hannover, Germany; abuisneineh.rene@mh-hannover.de

**Keywords:** hypoxemia, oxygen therapy, functional outcome, emergency admission

## Abstract

**Highlights:**

**What are the main findings?**

- Significant functional deterioration occurs in the majority of patients after hospital admission due to acute hypoxemia;
- Patients with acute hypoxemia are usually elderly and the leading underlying diseases are infections, COPD exacerbation and congestive heart failure;

**What is the implication of the main finding?**

- In a planned randomized controlled trial to study permissive hypoxemia, there should be an upper age limit and patients with metastatic malignancy should be excluded.

**Abstract:**

Background: Many hospitalized patients decline in functional status after discharge, but functional decline in emergency admissions with hypoxemia is unknown. The primary aim of this study was to study functional outcomes as a clinical endpoint in a cohort of patients with acute hypoxemia. Methods: A multicenter prospective observational study was conducted in patients with new-onset hypoxemia emergently admitted to two respiratory departments at a university hospital and an academic teaching hospital. Using the WHO scale, the patients’ functional status 4 weeks before admission and at hospital discharge was assessed. The type and duration of oxygen therapy, hospital length of stay and survival and risk of hypercapnic failure were recorded. Results: A total of 151 patients with a median age of 74 were included. Two-thirds declined in functional status by at least one grade at discharge. A good functional status (OR 4.849 (95% CI 2.209–10.647)) and progressive cancer (OR 6.079 (1.197–30.881)) were more associated with functional decline. Most patients were treated with conventional oxygen therapy (n = 95, 62%). The rates of in-hospital mortality and need for intubation were both 8%. Conclusions: Patients with acute hypoxemia in the emergency room have a poorer functional status after hospital discharge. This decline may be of multifactorial origin.

## 1. Introduction

Hypoxemia refers to arterial blood in which the amount of oxygen (partial pressure) or oxygen saturation is decreased. Commonly, the thresholds of partial pressure of oxygen (PaO_2_) less than 60 mmHg and oxygen saturation (SaO_2_) less than 90% in arterial blood are used. In contrast, hypoxia refers to an inadequate oxygen supply to organs and tissues [1]. In clinical medicine, detecting hypoxemia is crucial as it serves as an alarming sign requiring immediate attention. In emergency situations, pulse oximetry is commonly used to identify hypoxemia. In the acute management of hypoxemia, oxygen therapy is the mainstay of initial therapy.

The precise range of hypoxemia tolerated over an extended period in adults remains uncertain due to the absence of controlled trials in adults. Consequently, the question of how low the level of hypoxemia that can be sustained is cannot be definitively answered based on current knowledge.

In the absence of randomized trials on oxygen therapy in patients with acute hypoxemia admitted to emergency care, the impact of oxygen therapy on survival and other patient-relevant outcomes remains unclear.

There is limited knowledge about the incidence of functional decline in emergency admissions due to hypoxemia. In a recent randomized controlled trial, 27% of COVID-19 patients hospitalized with respiratory failure had a functional decline by day 28 [2]. Studies usually focus on long-term functional decline after discharge in general without regard for the role of hypoxemia [3].

Data showing mortality and intubation rates in hypoxemic patients differ depending on the underlying disease. Mortality 28 days after admission was reported to be 8% in hypoxemic patients with community-acquired pneumonia and COPD exacerbations [4,5]. The need for intubation within 28 days was reported to be 8% in the same patient groups [5,6].

To assess functional decline in hospitalized patients with acute hypoxemia, observational data are limited despite growing attention to oxygen use in German hospitals [7].

The aim of this study was to study functional decline according to the WHO scale as a clinical endpoint in a cohort of patients with acute hypoxemia as part of a planned national clinical trial comparing different oxygen targets. The second aim of this trial was to identify the appropriate inclusion criteria and outcome variables to calculate a sample size for such a trial.

## 2. Materials and Methods

This prospective observational study was performed in two hospitals (a 1500-bed university hospital and a 560-bed academic teaching hospital) in Hannover, Germany. This study was conducted following the ethical guidelines of the 1975 Declaration of Helsinki and was approved by the local ethics committee (No 10329_BO_K_2022). It was registered in the German registry of clinical studies (DRKS00032358, functional decline in hospital admission with acute hypoxemia (FUNDOX)). Patients signed informed consent forms before inclusion. 

Adult medical patients with new-onset hypoxemia admitted via the emergency department were included. Hypoxemia was defined as first-recorded oxygen saturation measured by pulse oximetry (SpO_2_) < 90% without oxygen therapy (O_2_) on admission to the emergency department or in the ambulance or the need for oxygen supplementation. Key exclusion criteria were prior long-term oxygen therapy (LTOT); patients using supplemental O_2_ intermittently during, e.g., exertion; first-recorded SpO_2_ > 96% with oxygen therapy on admission or in ambulance; need for invasive mechanical ventilation; and lack of informed consent. Patients were followed until hospital discharge. 

The primary aim of this study was to observe the functional outcomes of hospital admissions with acute hypoxemia at hospital discharge. Functional status 4 weeks prior to admission was recorded retrospectively on admission; it was either discussed with the patient in person or, if the patient was unable to provide adequate information, relatives were asked. Functional status at hospital discharge was recorded prospectively. Functional status was graded according to the WHO scale. Briefly, on this scale, patients were graded as 0 if they were fully active and able to perform all pre-disease activities without restriction; as 1 if they were restricted in physically strenuous activities but ambulatory and able to perform light or sedentary work, such as light housework; as grade 2 if ambulatory and able to perform all self-care activities but unable to perform any work activities; up to and about >50% of waking hours; as grade 3 if able to provide only limited self-care, and confined to bed or chair > 50% of waking hours; and grade 4 if totally disabled and unable to perform any self-care activities, and totally confined to bed or chair. Grade 5 in the follow-up was death of the patient [8]. 

Ventilation time was calculated in days from time of intubation to extubation or decannulation in the case of tracheostomy.

The calculation determined the proportion of patients who experienced functional deterioration, defined as a decline in at least one stage on the WHO scale. Additionally, this study recorded the need for intubation, the need for high-flow oxygen therapy (HFNC), non-invasive ventilation, hospital length of stay, hospital survival, duration of oxygen therapy, and LTOT on discharge were additionally recorded in patients. 

The oxygen content (CaO_2_) was calculated using the formula SpO_2_ × Hemoglobin (g/dL) × 1.34. Hypercapnic respiratory failure is defined as the partial pressure of carbon dioxide (paCO_2_) > 45 mmHg in arterial or capillary blood gas analysis. The risk for hypercapnic failure is defined as a diagnosis of COPD, obesity with a body mass index > 40 kg/m^2^, emergency admission for cystic fibrosis or asthma, or in adults > 35 years with neuromuscular disease or scoliosis [9]. 

Sample size calculation: The estimated decline in functional status was expected to occur in 30% of patients. Given a probability of type I error (alpha 0.05) and a power of 80% the required sample size was n = 137, with an estimated dropout rate of 10% [10]. 

Statistical analysis: Metric variables were presented as medians and 25 and 75% quartiles, while categorical variables were presented as absolute numbers and percentages of data entries. Ventilator-free days were expressed as a mean with standard deviation due to low intubation rates. Univariate analyses were conducted using the median test for continuous variables and the chi-square test or Fisher’s exact test for categorical variables. Survival analysis was performed using the Kaplan–Meier method. The last observation was the day of discharge or death of the patient. Binary logistic regression analyses were conducted to identify factors associated with decline in performance status. The level of significance was set at <0.10 to include variables identified by univariate analysis between groups. No imputation for missing data was performed. Variables with a proportion of >30% missing were excluded.

## 3. Results

During the nine-month study period between 11 April 2022 and 4 January 2023, 153 patients were included. The last patient was discharged on 20 March 2023. Patient demographics are displayed in Table 1.

The majority of patients admitted with hypoxemia were elderly with a median of 74 years and 51% had a previously good performance status (WHO grade 0 and 1). Forty-five patients (29%) were admitted due to a respiratory infection, 22 (14%) because of COPD exacerbation, 43 (28%) due to decompensated congestive heart failure and 17 (11%) because of cancer progression. 

One hundred and forty-three (94%) had at least one comorbidity. A total of 48 (31%) had a pre-existing diagnosis of COPD, 43 (28%) had been previously diagnosed with congestive heart failure, 29 were diabetic (19%) and 26 (17%) had a medical history of malignancy. Fifty-eight (38%) patients were at risk for hypercapnic respiratory failure, with forty-eight of them having a prior diagnosis of COPD. 

### 3.1. Treatment

**Figure 1:**
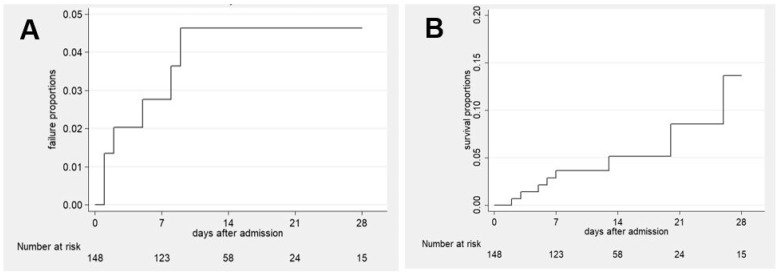
Kaplan–Meier failure graphs within 28 days: (**A**) time to intubation, (**B**) time to death.

In 115 (75%) patients, O_2_ was already administered in the ambulance with a median flow of 3 L/min; in the rest, oxygen therapy was initiated in the emergency department. A total of 58 (38%) patients had their SpO_2_ levels recorded without oxygen, with a median SpO_2_ of 87% (25, 75% percentile: 81, 88%). The majority of patients (n = 95, 62%) received conventional oxygen therapy, n = 17 (11%) with HFNC and n = 29 (19%) with non-invasive ventilation. Forty-one (27%) patients required treatment in the intensive care unit (ICU), and twelve (8%) were intubated. The Kaplan–Meier failure curve of intubation within 28 days is shown in Figure 1A. The median duration of oxygen therapy was 12 (25, 75% percentile 7, 18) days, respectively.

In 50 patients (33%), hypercapnic respiratory failure was confirmed in blood gas analysis, and 31 out of 58 patients (62%) were at risk (Table 2).

### 3.2. Outcome

The median length of hospital stay was 12 days (25, 75% percentile 8, 18). Twelve patients (8%) died in the hospital, with a median of 17 days (minimum 2, maximum 126 days) after admission. The Kaplan–Meier failure curve of death is displayed in Figure 1B. Three patients (2%) died after day 28 in the hospital (days 45, 63 and 126). Eight (5%) patients died without being intubated, of which three had progressive cancer, and five (3%) patients died without being treated in the ICU. Four out of twelve ventilated patients died in the hospital.

### 3.3. Performance Status at Discharge

**Figure 2:**
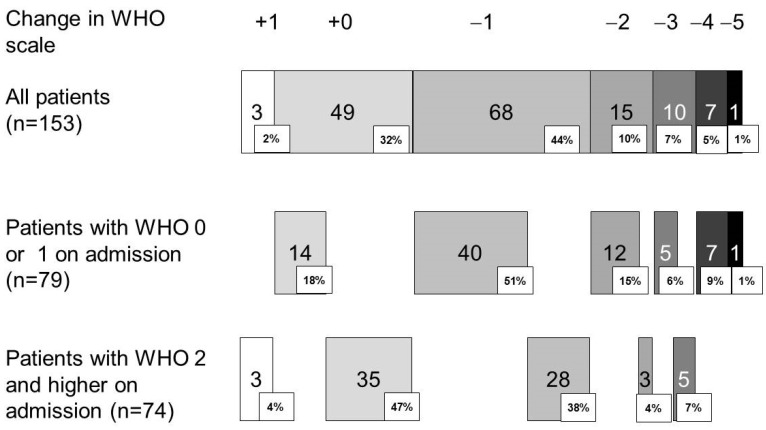
Change in WHO performance scale between 4 weeks before admission and hospital discharge. WHO: World Health Organization.

The majority of patients (135 out of 153, 82%) had a WHO performance status > grade 1 at discharge, rendering them unable to perform any work activities. One hundred and one patients (66%) had a decline in their WHO performance status by at least one grade at discharge, with a more pronounced effect in patients with a good performance status on admission (Figure 2). In the multivariate analysis (Table 3), a good performance at admission (WHO grade < 2, odds ratio 4.849, 95% confidence interval 2.209–10.647) and admission for progressive cancer (odds ratio 6.079, 95% confidence interval 1.197–30.881) were associated with functional decline at discharge.

Conventional oxygen therapy (n = 80) was not found to be associated with a reduced functional outcome in comparison to HFNC, NIV or mechanical ventilation (n = 73) (*p* = 0.31). 

Forty-four patients (29%) were discharged on oxygen treatment, with 50% of the patients with COPD exacerbation and progressive cancer being discharged on oxygen treatment.

## 4. Discussion

This study demonstrates that new-onset hypoxemic respiratory failure significantly impacts functional decline at hospital discharge in two-thirds of patients. 

Most of this functional decline may be attributed to underlying diseases rather than hypoxemia itself. Hypoxemia can be caused by various conditions, and functional decline and a persistent need for oxygen after discharge depend on underlying causes. Patients with malignant disease had a disproportionately high rate of functional decline, which contributed to their underlying disease. Conversely, patients with congestive heart failure had a disproportionately lower incidence. It is unknown whether correcting hypoxemia through oxygen therapy will improve patient outcomes. Unfortunately, there are no randomized controlled trials in adult patients studying the concept of permissive hypoxemia (SpO_2_ < 88%). Permissive hypoxemia did not affect mortality and functional outcome in randomized controlled studies using permissive hypoxemia in early-term newborns and small children [11,12].

In addition to patients with cancer, it has also been demonstrated that patients with good functional status upon admission were more likely to experience a decrease in functional status upon discharge. Patients admitted with a better initial status naturally have a greater potential to worsen their functional status than someone already admitted with a poor status. For instance, a patient with an initial status of WHO 1 can lose up to four points, whereas someone with a status of 3 can only lose two points.

One-third of our patients had COPD as a comorbidity, and every six patients were hospitalized for an exacerbation of the same. COPD is known to lead to reduced physical activity in daily life [13]. In addition, half of the COPD patients were discharged on long-term oxygen therapy. The duration of daily LTOT therapy is a determinant of both reduced daily physical activity and worse functional status, including fatigue and reduced exercise capacity [14]. According to the literature, in a retrospective Danish analysis of almost 15,000 COPD patients between 2001 and 2010, 76 to 91% of patients treated in the hospital for exacerbation were discharged on LTOT. LTOT could be discontinued within 6 months in 25% of the patients [15]. Information about LTOT discontinuation was not recorded during the follow-up in our study. According to German guidelines on both oxygen in acute care and LTOT, all patients were instructed to follow LTOT and it was mentioned in the discharge report that it should be followed to re-evaluate the indication for LTOT [9,16]. 

Following on from the topic of COPD, fifty patients (33%) were admitted with hypercapnic failure, and twenty-six of those patients had COPD. In total, 22 out of 48 COPD patients were admitted due to an exacerbation, with the majority being GOLD stage 3 and 4 patients. In these patients, hypoxemia, as well as hypercarbia, are frequent, while chronic hypoxia is usually preceding hypercapnia [17]. Although the majority of COPD patients do not need domiciliary oxygen according to national LTOT guidelines [9], most require very low-flow oxygen at rest to maintain adequate oxygenation. It can be concluded that those patients admitted with hypercapnia and COPD may already be chronically hypoxemic before admission. In the emergency room, it was not possible to differentiate between the acute or chronic condition in COPD patients.

Twenty-nine per cent of patients in our study were hospitalized due to pneumonia. In a recent analysis of 1054 cases of hospitalized patients with community-acquired pneumonia, the mortality rate within 30 days was 23% higher in elderly patients [18]. Similar to these findings, in our cohort, 10 patients had a primary diagnosis of community-acquired pneumonia and 2 died in hospital.

In elderly patients with pneumonia, frailty was found to be a strong determinant of lack of recovery in functional status six months after discharge due to hospitalization for pneumonia. The more frail the patients were before the acute event, the worse the outcome. Frailty before the acute event had a greater influence than disease severity [19]. 

Our study cohort represented a geriatric population with a median age of 74 years. Elderly patients in general have been shown to lose functionality during a hospital stay, subsumed under the term “hospitalization-associated disability” [3]. Functional decline is reported in 30–50% of discharged patients compared to their pre-illness baseline and is a predictor of adverse outcomes [20,21]. An older age and illness severity, particularly frailty, were predictors for prehospital and in-hospital functional decline [3]. Collecting functional status 4 weeks prior to hospital admission in our study, bias due to this acute prehospital loss of function is unlikely.

Presumably, the deterioration in the performance status after hospital discharge is a mixed picture of a wide variety of factors and is not solely caused by hypoxia, although it may contribute. In studies, the functional status of chronically ill or older patients is mostly observed without taking the role of hypoxia into account. Older, comorbid individuals are at a higher risk of developing pneumonia, COPD, and heart failure. Reduced physical activity during hospital stays further decreases functionality. The role of physiotherapy is unknown in our data and interventions to increase physical activity may be beneficial. 

A further, more profound, explanation for the loss of functionality lies in the effects hypoxia contributes to ageing processes and consecutively the functional decline and degenerative processes. During hypoxia, hypoxia-inducible factors (HIFs) are induced. HIFs are transcript factors which induce a variety of genes affecting, among other things, cell proliferation and metabolism [22]. This leads to impaired mitochondrial and cellular senescence [23]. Impaired mitochondrial function may lead to muscle atrophy, which in turn promotes physical inactivity leading to a decline in functional status [24].

Unfortunately, most of the studies focus on older patients with data missing regarding younger patients. In our study, mortality did not significantly differ between age tertiles and was 9, 4 and 11% in groups aged <65, 65–80 and >80 years (*p* = 0.342). 

The fact that two-thirds of those patients died without intubation points towards advanced palliative care planning in these patients. Therefore, there may be a bias in favor of increased mortality in our cohort due to the disproportionate share of palliative patients. Only four patients died while intubated during intensive care treatment. Similar results to ours could be found in two studies [4,5]. In a randomized study of 1058 hypoxemic patients (median age 78 years) with cardiac pulmonary edema, the 30-day mortality rate was 16% with only 3% of patients being intubated. This points to the fact that advanced care planning including withholding of intubation is a clinical reality in a geriatric population [25].

In our cohort, conventional oxygen therapy was favored (62%), followed by NIV (19%), HFNC therapy (11%) and mechanical ventilation (8%). Of those intubated, two-thirds survived.

NIV was preferred over HFNC in our study. The treating physicians had discretion over the therapeutic decision. HFNC appears to be not inferior to NIV, at least in moderate hypercapnia, in terms of intubation rate after 72 h [9]. The increased use of NIV may be explained by the large proportion of COPD patients. 

Some limitations of this study need to be considered. The results are limited to medical patients only. The patients did not receive follow-up after discharge and any recovery or discontinuation of LTOT could not be documented. This limits the understanding of long-term functional outcomes and recovery patterns. Room air SpO_2_ was recorded in only 58 patients. Supplemental O_2_ was recorded for the other 95 patients. To ensure that these patients were still truly hypoxic, SpO_2_ was not allowed to exceed 96%. This upper limit indirectly acted as an exclusion criterion for patients with oxygen being used to relieve dyspnea. Furthermore, this threshold was recommended in national guidelines [9] to prevent hyperoxia. Functionality was assessed verbally using a WHO scale, which, while standardized and straightforward, may not fully capture the complexity of functional status. A more differentiated assessment would be possible using instruments such as the Activities of Daily Living (ADL) index [21] or even the Clinical Frailty Scale (CFS), which should be incorporated in future studies rather than the WHO scale. It seems that the proportion of palliative patients is very high here so the results are more applicable to the geriatric population.

## 5. Conclusions

Although research has shown that hypoxemic patients are less functional after discharge than before, the sole effect of hypoxia is unclear. A future randomized control trial with a permissive hypoxemia trial may answer this question. In such a trial, an upper age limit and the exclusion of patients with metastatic cancer are encouraged. 

## Figures and Tables

**Table 1:** Patient characteristics.

	Hospitalized Patients with New-Onset Hypoxemia n = 153
Sex, n (%)	
Male	73 (48)
Female	80 (52)
Age on admission, median years (25, 75% percentile)	74 (62, 83)
Center, n (%)	
Urban hospital	123 (80)
University Hospital	30 (20)
WHO performance status at admission, n (%)	
0	28 (18)
1	51 (33)
2	54 (35)
3	12 (8)
4	8 (5)
Domiciliary oxygen, n (%)	0
Risk of hypercapnic failure, n (%)	58 (38)
COPD	48 (31)
Cystic fibrosis	4 (3)
Body mass index > 40 kg/m^2^	5 (3)
Neuromuscular disease	1 (1)
Scoliosis	2 (1)
COPD GOLD stage available on admission, n (% of all COPD)	18 (38)
COPD Stage 3 or 4	15 (83)
Oxygen supplementation in ambulance, n (%)	115 (75)
Oxygen flow used, median L/min (25, 75% percentile)	3 (2, 4)
Oxygen saturation available without oxygen supplementation, n (%)	58 (38)
Oxygen saturation without oxygen supplementation, median % (25, 75% percentile)	87 (80, 88)
Oxygen saturation with oxygen supplementation, median % (25, 75% percentile)	94 (92, 96)
Hemoglobin on admission, median g/dL (25, 75% percentile)	10.6 (12.9, 14.2)
Oxygen content on admission, median g/dL (25, 75% percentile)	14.2 (11.6, 16.0)

COPD—chronic obstructive pulmonary disease; WHO—World Health Organization.

**Table 2:** Treatment and outcome.

	Hospitalized Patients with New-Onset Hypoxemia n = 153
Hypercapnic failure (paCO_2_ > 45 mmHg), n (%)	50 (33)
Hypercapnic failure in patients at risk (n = 58)	31 (62)
Hypercapnic failure in COPD patients (n = 48)	26 (52)
Hypercapnic failure in COPD GOLD 3 & 4 patients (n = 15)	7 (47)
Admission to intensive care unit, n (%)	41 (27)
Admission to intermediate care unit, n (%)	12 (8)
Level of respiratory support *, n (%)	
High-flow oxygen	17 (11)
Non-invasive mechanical ventilation	29 (19)
Invasive ventilation	12 (8)
Extracorporeal support	0
Length of stay, median days (25, 75% percentile)	12 (8, 18)
Duration of oxygen therapy, median days (25, 75% percentile)	12 (7, 18)
Duration of invasive mechanical ventilation, median days (25, 75% percentile)	18 (4, 27)
Ventilator-free days at day 28, mean days ± standard deviation	26.3 ± 6.4
Initiation of long-term oxygen therapy at discharge, n (%)	44 (29)
WHO performance status at hospital discharge, n (%)	
0	2 (1)
1	26 (17)
2	56 (37)
3	41 (27)
4	16 (11)
Performance deterioration (≥1 WHO class, incl. death), n (%)	101 (66)
Hospital death, n (%)	12 (8)
Death after admission, median days (25, 75% percentile)	17 (7, 26)

COPD—chronic obstructive pulmonary disease; WHO—World Health Organization; paCO_2_—partial pressure of carbon dioxide. * combinations are possible.

**Table 3:** Group comparison and multivariate binary logistic regression analysis to predict a decline in performance status.

Covariate	Group Comparison		Univariate *p*-Value	Multivariate Analysis	
	Patients with Functional Decline n = 101	Patients without Functional Decline n = 52		Odds Ratio (95%-Confidence Interval)	*p*-Value
Female sex, n (%)	52 (51)	28 (54)	0.782		
Male sex, n (%)	49 (49)	24 (46)			
Age, median years (25, 75% percentile)	72 (62, 83)	77 (67, 82)	0.231		
WHO scale < 2 on admission, n (%)	36 (36)	38 (73)	**<0.001**	**4.849 (2.209–10.647)**	**<0.001**
WHO scale 2 to 4 on admission, n (%)	65 (64)	14 (27)			
Reason for admission, n (%)					
Respiratory infection	33 (33)	12 (27)	0.217		
COVID-19	10 (10)	3 (6)	0.385		
COPD exacerbation	13 (18)	9 (17)	0.459		
Progressive cancer	15 (30)	2 (4)	**0.040**	**6.079 (1.197–30.881)**	**0.030**
Congestion heart failure	23 (23)	20 (39)	**0.041**	0.896 (0.395–2.035)	0.794
No comorbidity, n (%)	6 (6)	3 (6)	0.966		
Any comorbidity, n (%)	95 (94)	48 (94)			
COPD (in n = 18 staging available)	33 (33)	15 (29)	0.629		
COPD Stage 3 or 4	6 (6)	9 (18)	0.017		
Congestive heart failure	26 (26)	23 (44)	**0.020**		
Diabetes	20 (20)	9 (17)	0.709		
Malignancy	20 (20)	6 (12)	0.197		
pCO_2_ > 45 mmHg	29 (29)	21 (40)	0.145		
Risk for hypercapnic failure, n (%)	40 (40)	18 (35)	0.547		
Cystic fibrosis	4 (4)	0	0.300		
COPD	33 (33)	15 (27)	0.629		
Body mass index > 40 kg/m^2^	3 (3)	2 (4)	1.000		
Neuromuscular disease	0	1(1)	1.000		
Scoliosis	0	2 (4)	0.114		
Admission to intensive or intermediate care	19 (19)	3 (6)	**0.029**	2.314 (0.464–11.546)	0.306
Level of respiratory support, n (%)					
High-flow oxygen	16 (16)	2 (4)	**0.034**	2.326 (0.352–15.368)	0.381
Non-invasive mechanical ventilation	20 (20)	9 (18)	0.689		
Invasive mechanical ventilation	9 (9)	3 (6)	0.752		

CI—confidence interval; numbers in bold indicate a *p*-value of <0.05.

## Data Availability

Anonymized participant data will be made available after publication upon requests directed to the corresponding author. Proposals will be reviewed and approved by the investigators and collaborators on the basis of scientific merit.

## References

[1] Considine J. (2005). The reliability of clinical indicators of oxygenation: A literature review. Contemp. Nurse.

[2] Ospina-Tascón G.A., Calderón-Tapia L.E., García A.F., Zarama V., Gómez-Álvarez F., Álvarez-Saa T., Pardo-Otálvaro S., Bautista-Rincón D.F., Vargas M.P., Aldana-Díaz J.L. (2021). Effect of High-Flow Oxygen Therapy vs Conventional Oxygen Therapy on Invasive Mechanical Ventilation and Clinical Recovery in Patients with Severe COVID-19. JAMA.

[3] Fimognari F.L., Pierantozzi A., De Alfieri W., Salani B., Zuccaro S.M., Arone A., Palleschi G., Palleschi L. (2017). The Severity of Acute Illness and Functional Trajectories in Hospitalized Older Medical Patients. J. Gerontol. Ser. A.

[4] Chang C.L., Robinson S.C., Mills G.D., Sullivan G.D., Karalus N.C., McLachlan J.D., Hancox R.J. (2011). Biochemical markers of cardiac dysfunction predict mortality in acute exacerbations of COPD. Thorax.

[5] Cillóniz C., Liapikou A., Martin-Loeches I., García-Vidal C., Gabarrús A., Ceccato A., Magdaleno D., Mensa J., Marco F., Torres A. (2018). Twenty-year trend in mortality among hospitalized patients with pneumococcal community-acquired pneumonia. PLoS ONE.

[6] Austin M.A., Wills K.E., Blizzard L., Walters E.H., Wood-Baker R. (2010). Effect of high flow oxygen on mortality in chronic obstructive pulmonary disease patients in prehospital setting: Randomised controlled trial. BMJ.

[7] Fühner T., Gottlieb J., Joean O., Klooster M.P.V., Kayser M.Z., Valtin C., Ewen R., Golpon H. (2022). Eine Querschnittsuntersuchung zur Qualität der Sauerstofftherapie in drei deutschen Krankenhäusern. DMW Dtsch. Med. Wochenschr..

[8] Oken M.M., Creech R.H., Tormey D.C., Horton J., Davis T.E., McFadden E.T., Carbone P.P. (1982). Toxicity and response criteria of the Eastern Cooperative Oncology Group. Am. J. Clin. Oncol..

[9] Gottlieb J., Capetian P., Hamsen U., Janssens U., Karagiannidis C., Kluge S., Nothacker M., Roiter S., Volk T., Worth H. (2022). German S3 Guideline: Oxygen Therapy in the Acute Care of Adult Patients. Respiration.

[10] Rosner B.A. (2011). Fundamentals of Biostatistics, Brooks/Cole.

[11] Schmidt B., Whyte R.K. (2020). Oxygen saturation target ranges and alarm settings in the NICU: What have we learnt from the neonatal oxygenation prospective meta-analysis (NeOProM)?. Semin. Fetal Neonatal Med..

[12] Maitland K., Kiguli S., Olupot-Olupot P., Hamaluba M., Thomas K., Alaroker F., Opoka R.O., Tagoola A., Bandika V., The COAST Trial Group (2021). Randomised controlled trial of oxygen therapy and high-flow nasal therapy in African children with pneumonia. Intensiv. Care Med..

[13] Watz H., Waschki B., Boehme C., Claussen M., Meyer T., Magnussen H. (2008). Extrapulmonary effects of chronic obstructive pulmonary disease on physical activity. Am. J. Respir. Crit. Care Med..

[14] Mazzarin C., Kovelis D., Biazim S., Pitta F., Valderramas S. (2018). Physical Inactivity, Functional Status and Exercise Capacity in COPD Patients Receiving Home-Based Oxygen Therapy. COPD J. Chronic Obstr. Pulm. Dis..

[15] Ringbaek T.J., Lange P. (2014). Trends in long-term oxygen therapy for COPD in Denmark from 2001 to 2010. Respir. Med..

[16] Haidl P., Jany B., Geiseler J., Andreas S., Arzt M., Dreher M., Frey M., Hauck R.W., Herth F., Hämäläinen N. (2020). Guideline for Long-Term Oxygen Therapy—S2k-Guideline Published by the German Respiratory Society. Pneumologie.

[17] Abdo W.F., Heunks L.M. (2012). Oxygen-induced hypercapnia in COPD: Myths and facts. Crit. Care.

[18] Theilacker C., Sprenger R., Leverkus F., Walker J., Häckl D., von Eiff C., Schiffner-Rohe J. (2021). Population-based incidence and mortality of community-acquired pneumonia in Germany. PLoS ONE.

[19] Park C.M., Dhawan R., Lie J.J., Sison S.M., Kim W., Lee E.S., Kim J.H., Kim D.H. (2022). Functional status recovery trajectories in hospitalised older adults with pneumonia. BMJ Open Respir. Res..

[20] Gill T.M., Allore H.G., Holford T.R., Guo Z. (2004). Hospitalization, restricted activity, and the development of disability among older persons. JAMA.

[21] Covinsky K.E., Palmer R.M., Fortinsky R.H., Counsell S.R., Stewart A.L., Kresevic D., Burant C.J., Landefeld C.S. (2003). Loss of independence in activities of daily living in older adults hospitalized with medical illnesses: Increased vulnerability with age. J. Am. Geriatr. Soc..

[22] Yeo E.-J. (2019). Hypoxia and aging. Exp. Mol. Med..

[23] Zhang W., Feng Y., Guo Q., Guo W., Xu H., Li X., Yi F., Guan Y., Geng N., Wang P. (2020). SIRT1 modulates cell cycle progression by regulating CHK2 acetylation−phosphorylation. Cell Death Differ..

[24] Chen X., Ji Y., Liu R., Zhu X., Wang K., Yang X., Liu B., Gao Z., Huang Y., Shen Y. (2023). Mitochondrial dysfunction: Roles in skeletal muscle atrophy. J. Transl. Med..

[25] Gray A., Goodacre S., Newby D.E., Masson M., Sampson F., Nicholl J. (2008). Noninvasive ventilation in acute cardiogenic pulmonary edema. N. Engl. J. Med..

